# Key Roles of Dipterocarpaceae, Bark Type Diversity and Tree Size in Lowland Rainforests of Northeast Borneo—Using Functional Traits of Lichens to Distinguish Plots of Old Growth and Regenerating Logged Forests

**DOI:** 10.3390/microorganisms9030541

**Published:** 2021-03-05

**Authors:** Holger Thüs, Pat Wolseley, Dan Carpenter, Paul Eggleton, Glen Reynolds, Charles S. Vairappan, Gothamie Weerakoon, Robert J. Mrowicki

**Affiliations:** 1 Botany Department, State Museum of Natural History Stuttgart, 70191 Stuttgart, Germany; 2 Life Sciences Department, The Natural History Museum London, London SW7 5BD, UK; p.wolseley@nhm.ac.uk (P.W.); drdcarpenter@googlemail.com (D.C.); p.eggleton@nhm.ac.uk (P.E.); gothamie.weerakoon2@nhm.ac.uk (G.W.); robmro@mba.ac.uk (R.J.M.); 3 South East Asia Rainforest Partnership, Danum Valley Field Centre, Ladad Datu 91112, Malaysia; glen.searp@icloud.com; 4 Institute for Tropical Biology and Conservation, Universiti Malaysia Sabah, Kota Kinabalu 88400, Malaysia; csv@ums.edu.my; 5 Marine Biological Association, Plymouth, Devon PL1 2PB, UK

**Keywords:** lichenised fungi, forest assessment, forest degradation, Sabah, SAFE, Danum, Maliau

## Abstract

Many lowland rainforests in Southeast Asia are severely altered by selective logging and there is a need for rapid assessment methods to identify characteristic communities of old growth forests and to monitor restoration success in regenerating forests. We have studied the effect of logging on the diversity and composition of lichen communities on trunks of trees in lowland rainforests of northeast Borneo dominated by Dipterocarpaceae. Using data from field observations and vouchers collected from plots in disturbed and undisturbed forests, we compared a taxonomy-based and a taxon-free method. Vouchers were identified to genus or genus group and assigned to functional groups based on sets of functional traits. Both datasets allowed the detection of significant differences in lichen communities between disturbed and undisturbed forest plots. Bark type diversity and the proportion of large trees, particularly those belonging to the family Dipterocarpaceae, were the main drivers of lichen community structure. Our results confirm the usefulness of a functional groups approach for the rapid assessment of tropical lowland rainforests in Southeast Asia. A high proportion of Dipterocarpaceae trees is revealed as an essential element for the restoration of near natural lichen communities in lowland rainforests of Southeast Asia.

## 1. Introduction

Tropical forests are under threat from intensified land use and continue either to be altered or to disappear altogether at a high rate [1]. Efforts are made to select high priority sites for conservation as well as setting aside areas that have undergone some form of deterioration in the past but are now on a path to recovery [2]. Identifying high quality forest remnants and monitoring recovery in regenerating forests requires quick assessment methodologies in order to guide limited resources for conservation to the most valuable sites and to enable monitoring of the success of recovery in regenerating or newly established forests [3,4].

The diversity of lichenised fungi species and their associated functional traits decreases in disturbed forests across the world and in turn can be used to assess levels of disturbance [5,6]. Lichens are multi-organism systems [7,8] with usually one fungal taxon making up the majority of its heterotrophic biomass (and the name of that dominating fungus usually being used as the identifier for the entire lichen system), one or more photosynthetic components and various additional associated fungi and bacteria in minor quantities. Compared to other forest organisms, lichens have the advantage that they are visible to the naked eye all year round, and both fungal and algal or cyanobacterial components can be identified to at least some taxonomic level in the field. Functional traits and taxon composition in epiphytic lichen communities depend on factors such as micro- and macroclimate, structural and chemical habitat diversity as well as forest continuity with many of these factors being interconnected [8]. Studies on epiphytic lichen communities have allowed the separation of characteristic communities across macroclimatic gradients [9], between different degrees and types of disturbance [10,11,12,13,14,15], and in relation to different time scales of forest continuity [16,17,18].

Most attempts at classifying lichen communities in forested ecosystems for bio-indication purposes are based on the taxonomic recognition of their components [9]. In areas where the lichen biota are relatively well known, e.g., in Europe or N-America, an experienced specialist can carry out field identification of most lichens at species or genus level and only for a minority of taxa complementary checks in the lab are required in order to assess essential anatomical or chemical characters [19,20]. Temperate parts of the Northern Hemisphere have long been a focus of taxonomic work while tropical to temperate southern hemisphere areas continue to be comparatively less well studied. Even in temperate areas crustose lichens tend to be more difficult to identify in the field and they are included in routine lichen surveys of forest health only in the best known areas, e.g., in Europe [19,21], while guidelines in the USA exclude them [22].

Recent years have seen a surge in activities of lichenologists in tropical countries, but the number of local taxonomists remains far too low and the challenges from still undescribed and cryptic taxa too large, to allow species or even genus level assessment of lichen communities in tropical forests wherever it is needed. Furthermore, the field recognition of lichenised fungi in the tropics is hampered by the lack of comprehensive taxonomic treatments for many tropical parts of the world, the occurrence of taxa which cannot be separated without anatomical and/or chemical analysis in the lab [23,24]. This affects particularly wet lowland rainforests with little seasonality where morphological groups which are poor in characters directly visible in the field are often dominant, e.g., crustose species from the families Graphidaceae, Pyrenulaceae, Porinaceae and Arthoniaceae [14].

In the past, problems with the field identification of lichen species in the tropics were addressed, e.g., by analysing data at genus or family level [25]. Today however, genus delimitations of characteristic tropical families (e.g., Graphidaceae) follows much narrower concepts and often relies on anatomical characters, which are difficult or impossible to apply in the field [14]. In response to this challenge, methods were developed in the Neotropics which are based on morphological groups within a selected family (e.g., for thelotremoid Graphidaceae [26] or so called “biotipos” within selected genera [27]. However, the respective families or genera still need to be distinguished from other taxa which is particularly difficult for lowland rainforest lichens. Other authors abandoned the recognition of formally described taxa altogether in favour of growth forms alone to assess environmental change at large spatial scales within the same forest type [28] or microclimatic effects of forest fragmentation at the local scale [29]. None of these approaches have been trialled so far in tropical Southeast Asia.

Forest disturbance in Southeast-Asia is driven by a variety of factors, which differ depending on the specific region and landscape type. Lowland rainforests in particular are affected by the expansion of settlements, agriculture and silviculture (mainly for pulp timber), as well as various types of selective logging for the timber markets.

In Sabah (Northeastern Borneo) the use of forest products is regulated in the framework of Sabah Forestry Rules which date back to 1968 and have since been amended several times [30]. Lowland rainforests in this area have been affected mainly by selective logging with a focus on large Dipterocarpaceae trees and clear felling, followed by replacement with palm oil plantations.

Our study aims to test:

1. The separation of lichen communities in disturbed and undisturbed forest plots using datasets based on taxonomic identification (genus or genus-group) or by using morphological groups (“functional groups” defined by traits);
2. which elements of the forest structure differ between logged and undisturbed forest plots; and
3. whether changes in lichen diversity, from taxonomic and functional traits perspectives, are linked to specific changes in forest structure.

## 2. Materials and Methods

### 2.1. Study Area

Study sites were selected using a randomised approach in three areas of lowland forests in NE Borneo in the Malaysian state of Sabah. Two of them are examples of pristine rainforest, the Danum Valley Conservation Area (“D”) and Maliau Conservation Area (“M”). The third group of sites is located within the area of the Stability of Altered Forest Ecosystems Project (“S”), an area where industrial logging has created a secondary forest environment [31].

The main SAFE Project site (7076 ha) is part of a landscape of selectively logged forest to the north (part of the Ulu Segama Forest Reserve-Danum Valley complex of forests) and oil palm plantations and mostly highly degraded fragmented forest to the east, west and south. The SAFE area has been repeatedly logged over the past 40 years [32] with a final ‘salvage’ logging operation conducted between 2014 and 2017. Salvage logging removed all remaining commercial stems (other than from the experimentally created SAFE Project fragments and riparian reserves, which were not re-logged) as a pre-cursor to development as an oil palm plantation (Sabah Forestry Department, unpublished data). On initiation of the re-logging operation in 2014 a timber milling facility was established within the SAFE area and specifically equipped to process small diameter logs, mainly for the production of plywood veneers. Outside of the experimental fragments and riparian zones, the SAFE Project area consists of a highly degraded mosaic of open grassy areas, skidding tracks and collapsed roads and sparsely scattered remnant forest patches, confined mainly to slopes that were too steep to harvest.

**Figure 1:**
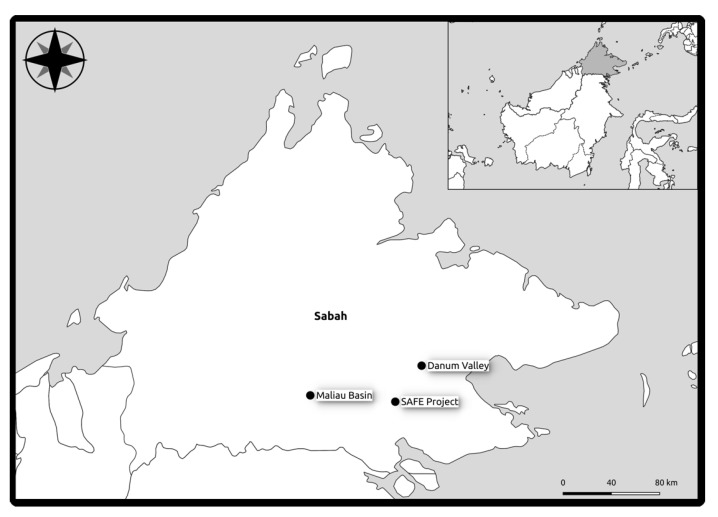
Map of study area and plot locations in Northeastern Borneo.

For Danum and Maliau the sampling areas were based on a 1 km square within an extensive primary lowland rainforest. In Danum and Maliau eight plots were located in each area along the x and y axes of this square at distances of 100 m, 200 m, 400 m and 800 m, with two plots located at 0 m. Plots were not located precisely as in the design due to the challenging terrain. The design accounts for the heterogenous nature of tropical forest and the variation in environmental conditions across relatively small distances. SAFE plots were located in each of the six 100 ha logged fragments that are part of the SAFE experimental design [31] (Figure 1).

In total we studied eight plots at each of Maliau and Danum and six at SAFE with 12 trees per plot (in total 264 trees).

Sabah has an average annual rainfall of 2630 mm per year [33] and annual average temperature of 26 °C (World Bank data). Local rainfall and relative humidity can vary substantially at the regional scale and are strongly influenced by topography, e.g., for two localities within the SAFE area rainfall varies between 2373 and 2619 mm per year. It is further influenced by forest cover as suggested by the difference of rainfall data between SAFE and a station at Danum with 2884 mm per year. Relative humidity (RH) was measured at Danum base camp at 8 and 14 h every day and could differ as much as 30% between readings where the 1st reading was >98% on most days. Temperature also varied greatly from 20–24 °C to 30–35 °C during a 24 h period [34]. The geology for Danum valley and the SAFE sites is mainly argillaceous rocks with some arenaceous and calcareous beds. The soils at Danum valley are orthic acrisol and dystric cambisols [35]. The soils at the SAFE project are mostly orthic acrisols, orthic luvisols, dystric and eutric cambisols and lithosols. Maliau’s geology is mainly arenaceous and argillaceous rocks with some chaotic deposits, and coal and calcareous beds. The soils of Maliau are orthic acrisols, dystric cambisols, gleyic podsols, humic gleysols and lithosols [35].

LiDAR (Light Detection and Ranging) data were used to characterise vegetation structure in the sample plots. Data were only available for the Maliau and SAFE plots for this study. LiDAR data were used to calculate tree canopy height (tch) and above ground biomass (agb). The Maliau plots had a mean canopy height of 35.94 m (*n* = 121, st dev = 7.64) and mean above ground biomass was 203.29 kg (*n* = 121, st dev = 69.18). The SAFE plots had a mean canopy height of 13.40 m (*n* = 928, st dev = 5.75) and mean above ground biomass of 42.74 kg (*n* = 928, st dev = 27.99).

### 2.2. Lichen Data

Tree selection and recording of lichen frequencies largely followed the BioAssess method [19] with frequency data collected from five 10 × 10 cm ladder quadrats placed on four principal aspects for 12 trees per plot.

Differences to the BioAssess sampling protocol were necessary due to the dominance of crustose taxa in our plots, with many of them in sterile condition. This renders some of them impossible to identify in the field, even at a generic or family level. We recorded morphological taxon groups in the field (Table S1), which we assembled based on easily observable traits and collected vouchers from each site in order to allow retrospective assignment of the field data to at least genus or genus group level, following more detailed studies of the collected material in the lab.

For data analysis we grouped observations of lichens with shared morphological traits of known or assumed relevance for the ecological performance of the lichen thalli (functional response traits, Tables S2–S3): (1) the associated photobiont (S3: Functional Trait class 3), (2) thallus shape (growth form; S3 FTc 1), (3) water repellent surface structures (S3 FTc 9-11), (4) pigmentation (S3 FTc 2) and (5) the type of dispersal structures (S3 FTc 4, 6, 7, 8).

(1) *Photobionts*

The most common photobionts in tropical lichens are species in the Chlorophyta families Trentepohliaceae and Trebouxiaceae and the two Cyanobacteria genera *Nostoc* and *Rhizonema* [36,37]. While mixed populations of different photobiont taxa can occur in the same thallus, usually these species belong to the same family, except for Cyanobacteria which can be present as secondary photobionts in specialised structures (cephalodia) of some Trebouxiophyceae associated lichens. Such secondary cyanobacterial photobionts appear to be generally absent in lichens with Trentepohliaceae as the main photobiont. While at least some of the lichens with Chlorophyta photobionts can achieve a positive carbon balance based on access to high air humidity alone, lichens with Cyanobacterial photobionts generally rely on access to liquid water [38,39]). We identified Cyanobacteria in the field by the typical blueish or brown colour of the photobiont cells which become visible by scratching the surface of the lichens when wet. Lichens with the two different most frequent Cyanobacterial photobiont genera show distinct differences in their water uptake and thallus structure. Both free living strains and lichenised *Nostoc* are capable of the development of large quantities of a gelatinous matrix. This gelatinous matrix causes lichen thalli associated with *Nostoc* (in our study area mostly Collemataceae) to swell up substantially when wet, thereby storing large quantities of water and delaying subsequent desiccation [38]. *Rhizonema*-associated lichens in our study area (= *Coccocarpia* spp.) swell up to a much lesser degree and the storage capacity of the lichen thalli is smaller. The different response of the thalli to water uptake can easily be observed already in the field and—together with the brownish to bluish colour of the photobiont layer—allows a recognition of these two cyanobacterial photobiont types.

Among Chlorophyta photobionts Trentrepoholiaceae is the dominant family in hot humid tropical climates [40,41]. Accurate genus level identification in the family often relies on molecular characters and cannot be achieved in the field or even by microscopic examinations of lichen tissue [42,43]. At the family level Trentepohliaceae algae can be easy to identify in some lichens, where a characteristic yellow to orange colour of the algae can be seen when the algal cells are exposed. This character however is not reliable enough for consistent identification, because in some lichens the colour of the Trentepholiaceae photobiont is a shade of green no different to those of other Chlorophyta and only microscopic checks reveal their true identity. Therefore, we only distinguished between *Nostoc*, *Rhizonema* and Chlorophyta as observable photobiont groups in the field. Due to the difficulties in field identification of some lichenised Trentepholiaceae phenotypes we excluded the association with this taxon for the assembly of functional groups in the proposed future quick assessment of forest conditions. All tropical lichens with lirellate or thelotremoid fruiting bodies (Graphidaceae incl. Thelotremataceae) and all Arthoniales so far tested, are constantly associated with Trentepohliaceae photobionts [44]. Although we did not record them in the field, we used the association with Trentepohliaceae as a trait in the data analyses based on literature references. Only for sterile crusts we kept this trait unspecified, because false negative observations in the field were expected.

(2) *Thallus Shape (Growth Form)*

Overall thallus shape or growth form affects light harvesting and water relations alike [45]. We follow the common distinction between crustose, squamulose, foliose and filamentous growth types [5,10,11], although the distinction between the first three are not clear cut and can differ even for individuals of the same species (e.g., in our study area the thalli of *Catarraphia* are usually crustose but under ideal conditions become squamulose eventually). The frequency of specific growth forms has been shown to be linked to different disturbance levels in tropical and temperate forests alike [9,13,25,46,47].

(3) *Hydrophobic Thallus Structures*

The amount and routes of water uptake in lichen thalli are influenced by hydrophobic structures which can be developed at the surfaces of lichen thalli and in their interior [48]). Hydrophobic elements are particularly common in lichens in tropical rainforest where the combination of high night temperatures and humidity results in a negative carbon balance due to high respiration rates during the night and gas diffusion limitations for photosynthesis during the day due to supersaturation in the thallus [45]. The amount of rain throughfall and the dynamics of humidity conditions in the forest interior is influenced by forest structure [29,45]. Forest disturbance can result in more open conditions, e.g., by increasing the length of forest margins through fragmentation, and/or thinning tree density, but in some cases, this can also result in shadier conditions if the disturbed forest is dominated by thick carpets of lianas and high densities of rapidly growing secondary forest tree species (e.g., *Macaranga*) [49]. Such changes can affect the frequency of functional traits such as strongly hydrophobic surfaces either associated with the prothallus, hypothallus, or in on the surface of cortex free lichens and particularly lichens with an entirely byssoid thallus which become more prominent in lighter conditions and generally along disturbed forest edges [29].

(4) *Pigments*

Experimental studies on the role of pigments for the ecological performance of tropical forest lichens are still missing, but scattered evidence suggests that bright colours are linked to successful establishment in light-rich habitats which in the case of lowland rainforests are often associated with disturbance. In the family Teloschistaceae the development of coloured Anthraquinones was shown to be a key innovation in the evolution of high illumination tolerant lineages and an adaptive radiation into open canopy habitats [50]. Similarly, brightly coloured (but Anthraquinone-free) species in the Candelariaceae are among the most tolerant lichens to high illumination levels [51]. Observations in tropical forests of Southeast Asia support a trend for brightly coloured pigmented lichens to be more frequent in open and disturbed habitats compared to shaded conditions of undisturbed old growth forest types with a densely closed canopy [25], e.g., in the Dipterocarp lowland rainforests in our study area located in Northeastern Borneo.

(5) *Dispersal Structures*

The presence or absence of fruiting bodies and principal types of fruiting bodies are easy to observe in the field, but little is known so far on the ecological significance of this trait, although many lichens with stalked mazaedioid fruiting bodies are generally seen as potential indicators of ecological continuity [52]. In the family Graphidaceae many subtypes of “thelotremoid” fruiting bodies have been described and used to define morphological groups as indicators of old growth forests [26], but the functional basis for the association of these fruiting bodies with undisturbed forests remains unclear. It may be that these fruiting bodies represent only morphological markers of groups of taxa which have evolved physiological adaptations to old growth forests, the visible marker may therefore not be the functional trait responsible for the poor performance of these lichens in disturbed habitats. The variation in life cycles may be important in lichen distribution so that vegetative propagation may occur early on and be more successful in disturbed habitats while sexual reproduction may occur later in the life cycle [25]. 

### 2.3. Environmental Data

The following functional traits were recorded for each tree (Table S4): (1) girth, measured at c. 1.5 m above ground level except for large trees with high buttresses where girth could only be estimated; (2) bark type (DR = dry thick, S = smooth thin, C = scaly, R = ridged/rough); (3) the presence of buttresses or flutes; and (4) dipterocarp vs. non-dipterocarp species. Additionally, for the majority of trees, (5) bark pH was determined from the mean of up to three readings, measured by a flat head-electrode in KCL following a protocol previously used in Thailand [14]). Trees were identified as far as possible with the help of local experts in Maliau (Kho Ju Ming) and Danum (Barnadus Bala Ola) in five out of 8 plots at Maliau and 7 out of 8 plots at Maliau. This was not possible in the SAFE plots where we only distinguished Dipterocarpaceae from other tree taxa and the same approach of a family level identification of trees was applied also to one plot in Danum and two in Maliau where we did not have confirmed genus or species level identifications from tree experts. To facilitate the calculation of plot level metrics encapsulating the diversity of tree characteristics (see below) mean pH was converted into a categorical variable with four levels based on quartiles calculated from the overall distribution of per-tree pH values).

### 2.4. Data Analysis

Data analyses were conducted at plot level, whereby plots rather than individual trees were treated as replicates. Thus, lichen taxonomic and functional group abundance data were summed separately for each plot.

Additionally, in order to enable plot level characterisation of tree traits as continuous variables, we calculated the Shannon-Wiener diversity (H’) of (1) bark type and (2) pH categories, and the proportion of (3) large trees (i.e., girth ≥ 150 cm, reflecting the apparent shift of the tree size spectrum of trees at the selectively logged site compared to pristine forest sites), (4) trees with buttresses or flutes and (5) dipterocarp trees within each plot.

Trees with particularly large girth and Dipterocarp trees in general were the priority targets during the early phase of selected logging which took place in NE Borneo for several decades, but while the extraction of the largest trees was mostly complete in forest sites without high protection levels the intensity of logging for dipterocarps with smaller girth was highly variable at small spatial scale [31,49]. We use the relative frequency of large trees and Dipterocarps per plot as indicators for the degree of past disturbance. We also wanted to test how far the extraction of these particular tree groups explains changes in lichen communities alone or if other more complex effects on the forest structure which we could not quantify during our field campaign (e.g., changes in undergrowth and light conditions) may have to be taken into account. Lichen taxonomic group richness (S), Simpson’s evenness (1−λ) and Shannon-Wiener diversity (H’) were calculated for each plot. Additionally, the following distance-based functional diversity indices were calculated from lichen functional group abundance data in conjunction with the functional traits matrix (Table S2), using the function *dbFD* in the R package FD (v. 1.0.12) [51]): functional richness (FRic), functional evenness (FEve), functional divergence (FDiv) and functional dispersion (FDis).

The community-level weighted means (CWMs) of lichen functional traits were also calculated for each plot, based on the summed abundances of trait classes (i.e., the product of the functional group abundance and functional trait classification matrices [13,53,54,55], via the *FD::functcomp* function.

Prior to multivariate analyses (PERMANOVA, SIMPER, BIOENV, dbRDA and CAP—see below), lichen taxonomic and functional group abundance data and trait CWMs were log10(x + 1)-transformed to reduce the influence of numerically dominant groups and trait classes [56].

Permutational analysis of variance (PerANOVA) was used to test for differences in lichen taxonomic and functional group richness and diversity among the three sites. Differences in multivariate lichen community structure among sites were tested using permutational multivariate analysis of variance (PERMANOVA), separately for taxonomic and functional groups. Models included the factor ‘site’ (fixed, 3 levels: D, M and S), with a planned comparison (D, M) vs. S (i.e., undisturbed sites vs. disturbed site). ‘Plot’ (nested within site) was also included a random factor to account for potential non-independence of trees located within the same plot. Where a significant effect of ‘site’ was identified, pairwise permutational post hoc tests were used to reveal differences between individual factor levels. Analyses involved 9999 permutations of residuals under a reduced model, and tests were based on Type II Sums of Squares owing to unequal numbers of observations among groups.

To complement these unconstrained multivariate analyses, overall patterns in lichen community structure were visualised using non-metric multidimensional scaling (MDS) ordination plots, based on both taxonomic and functional groups.

Relative contributions of individual lichen taxa or functional groups to differences in community structure between groups of sites identified by PERMANOVA were determined via similarity of percentages (SIMPER [57]). 

Univariate PerANOVAs were based on Euclidean distance matrices calculated from untransformed data. For all multivariate tests (both unconstrained and constrained, including dbRDA and CAP—see below) community dissimilarities were calculated using the Bray-Curtis coefficient.

The extent to which lichen taxonomic and functional groups, as well as functional traits, were associated with different combinations of sites was examined using extended Indicator Value analysis [58,59], via the *multipatt* function in the R package *indicspecies* (v1.7.9 [60]). Analyses were based on the ‘*IndVal*’ index [58], with the significance of associations tested using 9999 permutations.

To enable dissimilarities in lichen communities to be visualised in relation to tree functional traits, the five tree traits (=‘environmental’ variables) were used in a constrained distance-based redundancy analysis (dbRDA), a variant of canonical analysis of principal coordinates (CAP [61]), using the *capscale* function in the R package vegan (version 2.5-7 [62]).

This analysis was performed separately for lichen taxonomic and functional groups and trait classes, using summed lichen group abundances, trait CWMs and continuous tree trait variables calculated for each plot as described above. Euclidean distances were calculated from environmental data, which were *z*-standardised to account for scaling differences between variables.

Prior to plot-level analyses, pairwise Spearman rank correlations were used to assess the extent of collinearity among tree traits. Overall, although there appeared to be a moderate positive relationship between the proportions of large trees and dipterocarps within plots (ρ = 0.64), there were no strong correlations between bark type diversity and proportions of large trees, trees with buttresses/flutes and dipterocarp trees that might be expected to severely disrupt the interpretation of results (|ρ| ≤ 0.7 [63]), therefore all four variables were included in subsequent analyses (Table S5). The biota–environment (BIOENV [64]) routine was used to identify the optimal subset of environmental variables accounting for variability in lichen community structure, via maximisation of the rank correlation between environmental and biological distance matrices. The variables identified by the BIOENV analysis were then used in the dbRDA analysis. The resulting ordination plots incorporated vectors representing. Spearman rank correlations were calculated between continuous environmental variables and the first two CAP axes.

The significance of overall models (based on the sum of all eigenvalues) and of constraining variables (marginal terms) were assessed via ANOVA-like permutation tests involving 9999 permutations. Spearman rank correlation coefficients between individual taxonomic or functional group abundances and CAP axes were calculated to identify the most important groups (i.e., |ρ| ≥ 0.5) contributing to variability in lichen assemblage structure.

Further CAP analyses were used to estimate the accuracy with which plots may be assigned to sites based on lichen community structure. This cross-validation method involves the ‘leave-one-out’ approach of Lachenbruch and Mickey [65] to determine the proportion of individual observations (in this case, plots) that are successfully allocated to the correct group 2003 [61]. Using the *CAPdiscrim* function in the R package BiodiversityR (v. 2.11.3 [66]), this analysis was performed separately for lichen taxonomic and functional groups and trait CWMs, versus a single factor, ‘site’ (i.e., a discriminant analysis, rather than a canonical correlation analysis). The appropriate numbers of principal coordinate axes (m) involved in the discriminant analyses were selected automatically based on the minimum misclassification error.

Analyses were conducted in R (v. 4.0.3 [67]), except for distance-based PerANOVAs and PERMANOVAs, which were performed using the PERMANOVA+ add-on in PRIMER (version 6.1.13; PRIMER-E Ltd., Plymouth, UK).

## 3. Results

### 3.1. Tree Diversity and Forest Structure

Diversity of sampled trees included 77 species in 30 families of which 16 trees were species of Dipterocarpaceae in 5 genera contributing to >30% of the trees sampled. Within the SAFE plots only 4 Dipterocarpaceae trees remained contributing to 5.5% of trees sampled. Girths varied hugely in Danum and Maliau including giant trees > 300 cm girth but in the SAFE plots although sampled trees exceeded 100 cm girth no Dipterocarpaceae trees exceeded 100 cm girth, suggesting that selective logging included Dipterocarpaceae trees >100 cm girth (Table S4).

With the exception of pH diversity (*p* = 0.238; PerANOVA ‘(D,M) vs. S’ planned comparison), all plot-level tree functional trait metrics (bark type diversity and proportions of large trees, trees with buttresses/flutes and dipterocarps) were lower at the logged site compared to the two old growth sites (Figure S1; Table S5), although for the proportion of trees with buttresses/flutes, the overall effect of site was not significant (*p* = 0.130).

### 3.2. Lichen Diversity, Traits and Groups 

Lichen identifications were aggregated into 13 taxonomic groups (genera and genus groups, Table S2) of which by far the largest generic component in Danum and Maliau was *Eschatagonia* and crustose thelotremoid Graphidaceae. In SAFE it was *Cryptothecia*. In parallel we distinguished 14 functional groups of which the largest component in Danum and Maliau was squamulose lichens with a green algal photobiont and no hypothallus which now included species of *Eschatagonia* and *Flakea*. In SAFE it was crusts without a cortex and a fimbriate prothallus in the functional group, corresponding to species of *Cryptothecia* and other unidentified crusts in the taxonomic groups.

#### 3.2.1. Lichen Taxonomic and Functional Diversity

**Figure 2:**
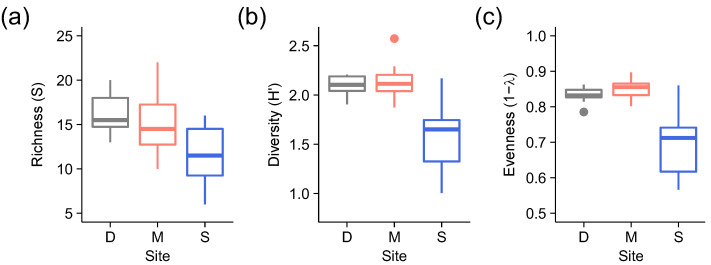
Boxplots of (**a**) richness (S), (**b**) diversity (H’) and (**c**) evenness (1 − λ) of lichen taxonomic group versus site (D = Danum Valley Conservation Area, M = Maliau Conservation Area [old growth sites]; S = Stability of Altered Forest Ecosystems [SAFE] Project area [logged site].

Lichen taxonomic diversity and evenness were significantly lower at the logged site (S) compared to the two old growth sites (D and M) (Figure 2; Table 1). For richness, although there was only a marginally significant effect of site (*p* = 0.052), the planned comparison between old growth and logged sites suggested that richness was greater at the former (*p* = 0.017). 

**Figure 3:**
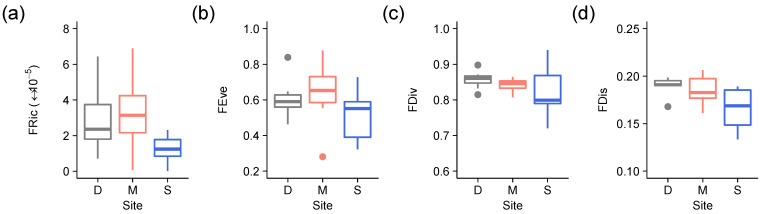
Boxplots of (**a**) functional richness (FRic), (**b**) functional evenness (FEve), (**c**) functional divergence (FDiv) and (**d**) functional dispersion (FDis) calculated for lichen functional groups versus site (D = Danum, M = Maliau [old growth sites]; S = SAFE [logged site]). Colour codes are grey for Danum, red for Maliau and blue for SAFE.

Of the four distance-based Functional Diversity indices, FRic and FDis were significantly lower at the logged site compared to the unlogged sites, as revealed by ‘(D,M) vs. S’ planned comparisons (FDis: *p* = 0.013; FRic: *p* = 0.038) (Figure 3; Table 2).

#### 3.2.2. Lichen Community Structure and Trait Composition

**Figure 4:**
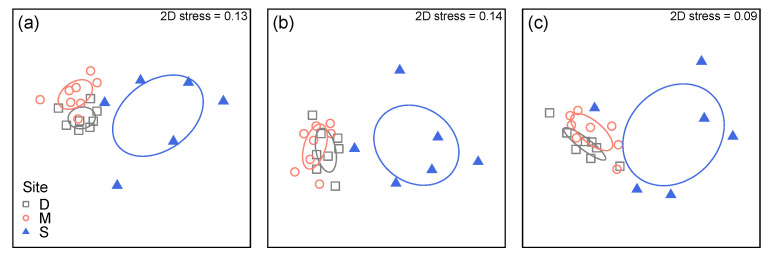
Non-metric multidimensional scaling (MDS) plot of lichen community structure as (**a**) taxonomic and (**b**) functional groups, and (**c**) functional trait composition, using ‘plot-level’ data summarised for each plot. Based on Bray-Curtis dissimilarities calculated from log10(x + 1)-transformed abundance data. Ellipses represent the standard deviation of points within each group.

There were highly significant differences in overall lichen community structure (in terms of both taxonomic and functional groups) and functional trait composition (as trait CWMs) between old growth and logged sites, but not between Danum and Maliau Conservation Areas (Table 3). This pattern was also evident from MDS plots showing strong separation between the SAFE Project area and the other two sites (Figure 4).

Of the lichen taxonomic and functional groups making the greatest contributions to differences in community structure were *Eschatagonia*, lirellate and thelotremoid Graphidaceae, and *Coccocarpia*. *Herpothallon*, *Leptogium*, *Micarea*, *Pyrenula*/*Anthracothecium* and *Phyllopsora/Krogia*. From a functional group perspective these groups correspond to squamulose lichens with a Chlorophyta photobiont with or without hypothallus, thelotremoid crusts, byssoid crusts with apothecia, crusts with lirellate apothecia, crusts with melanised perithecia, foliose cyanolichens with brittle thalli when dry, gelatinous when wet, foliose cyanolichens with robust thalli when dry and only little swelling when wet, crusts with mazaedioid apothecia and squamulose thalli with apothecia and hypothallus.

The opposite trend showed *Cryptothecia* sp., *Biatora* sp. and unidentified crusts under the taxonomic perspective and crusts with fimbriate prothallus, and apotheciate crusts, sterile crusts with fimbriate prothallus, sterile crusts with exposed pigments, and apothecioid crusts under the functional groups perspective.

The following lichen functional traits exhibited greater CWM values in old growth compared to logged sites: vegetative dispersal structures (i.e., soredia/isidia/pseudisidia), squamulose growth type, absence of fimbriate prothallus, non-byssoid surface structure, unknown Chlorophyta photobiont, apothecia with margins, and thelotremoid apothecia. 

Conversely, the following traits exhibited greater CWM values in the logged site: fimbriate prothallus, lack of sexual dispersal structures, byssoid surface structure, absence of vegetative dispersal structures, non-carbonised perithecia, crustose growth type, and Trentepohliaceae photobiont (Table 4).

Indicator Value analyses revealed a number of lichen taxonomic/functional groups and functional traits that were significantly associated with the combination of Danum and Maliau sites (i.e., old growth sites), rather than with each site separately; namely thelotremoid and lirellate Graphidaceae, Pyrenulaceae and *Coccocarpia* spp., or as functional groups “crustose thelotremoids”, “crustose lirellate”, “crustose perithecioid” and “foliose with little swelling when wet” and the corresponding individual traits of thelotremoid apothecia, lirellae, carbonised perithecia and a *Rhizonema* photobiont. Additionally, *Cryptothecia* spp. and the functional group of “crustose sterile crusts with fimbriate margin” were significantly associated with the logged site (Table S6).

The diversity of bark types and proportion of large trees and dipterocarps within plots were identified as key tree functional traits influencing lichen community structure, in terms of both taxonomic and functional groups (BIOENV; ρ = 0.60 and ρ = 0.65, respectively) and lichen trait classes (ρ = 0.60). In all three cases, CAP (dbRDA) axis 1 correlated negatively with all three plot-level tree traits, while axis 2 appeared to be related positively to bark diversity and negatively to the proportions of large trees and dipterocarps; the overall analysis was significant, as determined by permutation tests (taxonomic and functional groups: *p* < 0.001; trait classes: *p* = 0.004). However, none of the three environmental variables were significantly related either to lichen community structure or functional trait composition, as determined by marginal permutational tests.

**Figure 5:**
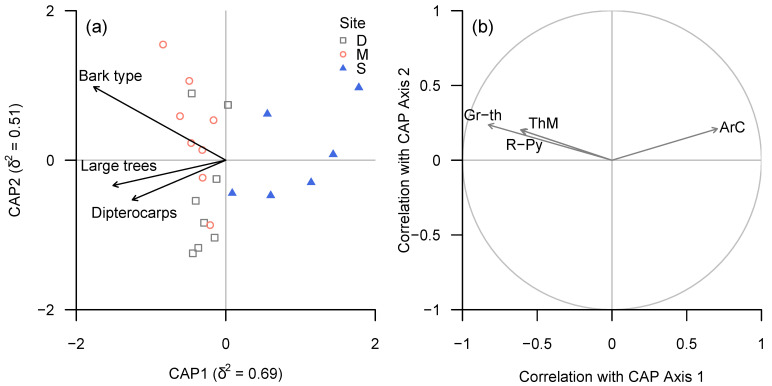
(**a**) CAP ordination plot of lichen community structure (taxonomic groups) according to continuous environmental (tree trait) variables, based on ‘plot-level’ data summarised for each plot. (**b**) Spearman rank correlation coefficients between CAP axes and individual taxa for which |ρ| ≥ 0.6. Taxonomic groups in (**b**): ArC—*Cryptothecia*, Gr-th—thelotremoid Graphidaceae, R-Py—*Phyllopsora* and *Krogia*, ThM—*Melanophloea*).

**Figure 6:**
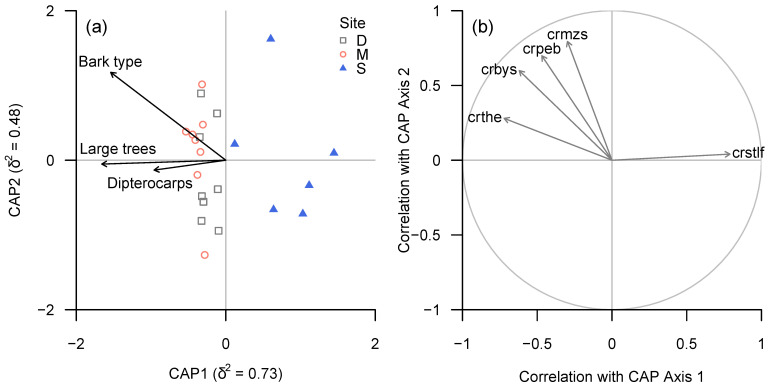
(**a**) CAP ordination plot of lichen community structure (functional groups) according to continuous environmental (tree trait) variables, based on ‘plot-level’ data summarised for each plot. (**b**) Spearman rank correlation coefficients between CAP axes and individual functional groups for which |ρ| ≥ 0.6. Functional groups in (**b**): crbys—crustose byssoid sterile, crmzs—crustose mazaedioid, crpeb—crustose perithecia melanised, crstlf—crustose sterile no cortex, prothallus fimbriate.

A large number of taxonomic groups and functional groups appeared to be associated strongly with plot-level bark diversity (Figure 5 and Figure 6).

Lirellate Graphidaceae (including a significant number of species new to science) and *Eschatagonia* spp. were associated with bark diversity, but also showed a strong association with the proportion of dipterocarps.

Conversely, *Cryptothecia* spp. and “unidentified sterile crusts” or (in terms of functional groups) “sterile crustose lichens with fimbriate margin” were negatively associated with both bark diversity and proportion of dipterocarps.

**Figure 7:**
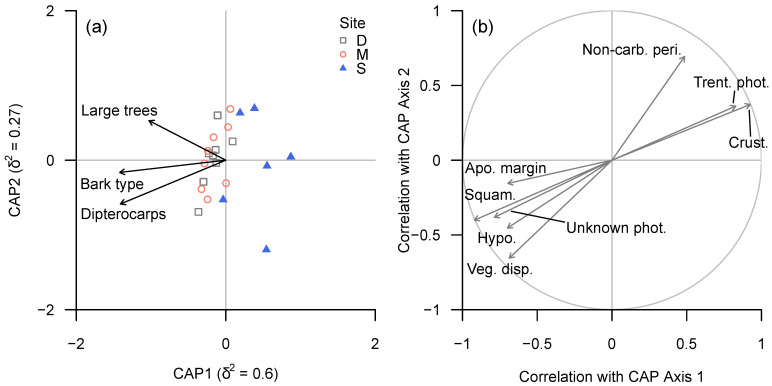
(**a**) CAP ordination plot of lichen functional trait composition according to continuous environmental (tree trait) variables, based on ‘plot-level’ data summarised for each plot. (**b**) Spearman rank correlation coefficients between CAP axes and individual functional groups for which |ρ| ≥ 0.6. Functional trait abbreviations: see Table 4c.

In terms of lichen traits, carbonised perithecia, apothecia without margins and thelotremoid apothecia were associated with bark diversity and, to a lesser degree, the proportion of dipterocarps; unknown Chlorophyta photobiont, presence of hypothallus, squamulose growth type and vegetative dispersal structures were associated more strongly with the proportion of dipterocarps. Crustose growth type, Trentepohliaceae photobiont, absence of vegetative dispersal structures and absence of hypothallus exhibited a strongly negative association with the proportion of dipterocarps (Figure 7).

The overall success with which plots could be classified by site based on lichen community structure and trait composition (CAP) was 68.18% (15/22 plots) for taxonomic groups, 72.73% (16/22 plots) for functional groups and 68.18% (15/22 plots) for trait classes.

## 4. Discussion

The use of a trait-based approach allows the inclusion of all lichen components in the data set of tropical forest samples, irrespective of identification of sterile and unidentified specimens which are a significant component in tropical communities. Although there is a loss of taxonomic information caused by employing an entirely taxon free approach (instead of at least genus/genus groups level identifications) for the assessment of similarity between Old Growth and Logged forest plots, the distinction between the two forest disturbance groups is still significant irrespective of the data type used.

Our results on functional diversity changes are nearly identical when functional diversity indices are calculated based on taxonomic groups or based on the data set with the taxon-free “functional groups” approach. Our results therefore support the use of functional traits based lichen groups as tools for the assessment of the disturbance level of lowland rainforests.

Significantly reduced functional richness and functional dispersion in the logged plots points to strong environmental filtering. This is probably due to the selective removal of large trees, and Dipterocarpaceae in particular, but may also be influenced due to differences in vegetative regeneration capacity after the logging and lack of seed trees in nearby areas in the case of Dipterocarpaceae. This was suggested by one plot within the SAFE area where large Dipterocarps were felled but never removed and several small er Dipterocarps remained, allowing some ecological continuity demonstrated in the similarity of lichen community structure with plots at Danum and Maliau in Figure 4. Functional evenness and Functional divergence instead do not change between the lichen communities from logged and unlogged plots in our study area, indicating that the occupancy of remaining ecological niches in the disturbed forest is similar to that of undisturbed forest. This means, that the current lichen community is limited by the range of micro-habitats in the disturbed forest and unlikely to change into the original condition over time by chance alone (e.g., by large distance dispersal from unlogged forest fragments). Only major structural changes within the now disturbed forest fragments (e.g., regrowth of large trees, particularly Dipterocarpaceae, and species with different bark types) will allow the re-establishment of a community truly similar to the undisturbed forest.

Logging in the disturbed plots has had the expected detrimental effects on the proportion of large trees, the proportion of Dipterocarps (of all size classes), but also on the diversity of bark types. Many lichens are more or less restricted to specific pH ranges of their substrata. The diversity of pH values however was not affected by the selected logging in our study areas.

Among the tested possible drivers of the separation between lichen communities in logged and unlogged forests bark type diversity (as a proxy to tree species diversity) turned out to be of high importance for most taxonomic and functional groups.

Bark of trees in tropical rainforests includes a great diversity of types from thin/smooth bark to thick/ridged bark characteristic of fire-resistant trees, and scaly barks which lose bark as they grow. It is not surprising though that species richness of trees is strongly linked to lichen diversity [68]. In tropical conditions where rainfall occurs heavily in short periods followed by high temperatures and lower RH the rate at which bark dries out strongly affects the lichen communities. Species with a cortex (“thelotremoid” Graphidaceae) maintained photosynthesis at lower RH than those without a cortex and byssoid thallus structure (*Herpothallon*) while filamentous (*Coenogonium*) and squamulose (*Phyllopsora*, *Krogia*) forms were most strongly affected by lower RH [69]). Where rainfall is very high throughout the year many lichens have hydrophobic compounds to prevent water logging and allow photosynthesis to continue [45].

Light conditions on trunks in a tropical rainforest are very low and this strongly affects macrolichens with a trebouxioid photobiont which cannot function efficiently in low light conditions [45]. Disturbed forests may have a lower canopy following selective logging, but the massive growth of vines casts a dense shade so that macrolichens with a trebouxioid photobiont are not a significant component and crustose lichens are the dominant component. In the canopy or in disturbed forests where conditions of temperature, UV and RH may vary over short periods of time thallus pigments are known to protect the photobiont from intense heat and UV to dry conditions [50].

In the logged areas sterile crusts are the single functional group with strongest dominance in most plots. Among the sterile crusts in the disturbed forest plots the low frequency of thalli with vegetative propagules (e.g., soredia, isidia, etc.) stands out. The apparent lack of both sexual and vegetative propagules in the thalli of many lichens in disturbed tropical forests was also reported from northern Thailand [17].

At the taxonomic level a high proportion of *Cryptothecia* spp. records is characteristic for the disturbed forest plots, together with many other sterile crustose taxa. Even the identification of *Cryptothecia* spp. at genus level can be challenging in the field, particularly when the thalli remain sterile. A taxonomy-based assessment of the disturbed forests is challenging here, because many thalli would require either chemical analyses or DNA-barcoding in order to quantify diversity and comparisons of species compositions. The functional traits approach has a clear advantage as it is based on field observations and includes the spectrum represented in the lichen community.

Some crustose groups where sexual reproduction is apparent present other problems particularly in the Graphidaceae where taxonomic changes are ongoing, and many new species are described every year [70]. Further identification of specimens from our plots in the lab showed that the 16 genera of Graphidaceae included several new species (pers. comm. R. Lücking). Previous work has shown that morphological groups defined by subtypes of thelotremoid fruiting bodies can be used as indicators of undisturbed old growth forests [26]. Our results support previous observation in Southeast Asia [71] and suggest that this is a general trend across the tropics. The separation of thelotremoid and lirellate fruiting bodies is clearly related to an ecological response to conditions in old growth forests. In other cases, fruiting bodies may be rare and identification to species of, e.g., *Phyllopsora* and *Krogia* impossible in the field, but the squamulose growth form is characteristic of the undisturbed rainforest.

While the sets of functional traits which we have used to distinguish unlogged old growth forest from heavily disturbed and secondary was specifically developed for lowland rainforests of northeast Borneo, the general methodology can be applied widely in other lowland rainforests of the tropics. In other climatic and geographic settings additional tests and calibration may be necessary in order to establish the response of lichens to their environmental conditions.

In a highly diverse tree flora that included 77 species in 30 families in Danum and Maliau, Dipterocapaceae represent the naturally dominant tree family in the study area, and they also include a large proportion of the trees which have the ability to grow to great sizes in this area. It is the large size which these trees can achieve in old growth forests and the general quality of their wood which place them among the first targets for selective logging, due to the high demand for this tree family on the timber markets [72].

The proportion of Dipterocarpaceae in a plot is a feature influencing specific taxonomic groups (*Eschatagonia* spp., lirellate Graphidaceae, *Micarea* spp.) more than others. These three groups are neither systematically closely related nor are those functional traits which we have studied here closely connected. For these groups, the link to Dipterocarpaceae trees may be via traits which we have not captured in our trait catalogue (e.g., physiological adaptations). Consequently, a loss of Dipterocarpaceae-dependent taxa may easily get overlooked in a functional groups approach based on external morphological characters when applied to forests with intermediate levels of disturbance. 

Our results show that the Dipterocarpaceae contribute to functional and taxonomic diversity of lichens in rainforests of northeast Borneo and that their presence is essential to allow the recovery of functional and taxonomic diversity in previously disturbed lowland rainforests in our study area. Natural regeneration of the tree species composition is unlikely due to large areas from which Dipterocarpaceae have been almost completely removed or depleted to the extent that recruitment fails. Targeted replanting of Dipterocarpaceae seedlings in the most degraded forests, and selectively tending naturally regenerating dipterocarp seedlings in situations where their survival and growth is impeded by competing vegetation, are likely to be crucial in the development of a near natural structure within a relatively short time-frame. 

## 5. Conclusions

Functional groups of lichens can be used to assess differences in forest quality in formerly logged and unlogged lowland rainforests of NE Borneo. 

In high quality sites, an assessment based on taxonomic units (genera or ideally species level) may capture a greater range of species associated with ecological niches of undisturbed sites, based on functional traits which are not yet known or insufficiently described. However, this requires specialist knowledge of a wide range of tropical lichen communities and environmental data that are not readily available.

Our study of epiphytic lichens on trunks of trees in lowland rainforests underlines the importance of Dipterocarpaceae in supporting lichen communities of Southeast Asian lowland forests and the need to address adequate representation of this family in forest recovery projects in this region.

## Figures and Tables

**Table 1:** PERANOVAs testing the effects of site (D = Danum, M = Maliau, S = SAFE) on (a) richness (S), (b) diversity (H’) and (c) evenness (1−λ) of lichen taxonomic groups, including planned comparisons between old growth (D, M) and logged (S) sites. Significant Monte Carlo *p* values are highlighted in bold.

		(a) Richness (*S*)			(b) Diversity (*H’*)			(c) Evenness (1–λ)		
Source of Variation	df	MS	Pseudo-*F*	*p*	MS	Pseudo-*F*	*p*	MS	Pseudo-*F*	*p*
Site	2	39.13	3.49	0.052	0.65	10.06	**0.002**	0.045	12.16	**<0.001**
(D, M) vs. S	1	74.25	6.84	**0.017**	1.29	20.78	**<0.001**	0.089	24.85	**<0.001**
Residual	19	11.22			0.065			0.0037		

**Table 2:** PERANOVAs testing the effects of site (D = Danum, M = Maliau, S = SAFE) on (a) functional richness (FRic), (b) functional evenness (FEve), (c) functional divergence (FDiv) and (d) functional dispersion (FDis) calculated for lichen functional groups, including planned comparisons between old growth (D, M) and logged (S) sites. Significant Monte Carlo p values are highlighted in bold.

		(a) FRic			(b) FEve			(c) FDiv			(d) FDis		
Source of Variation	df	MS	Pseudo-*F*	*p*	MS	Pseudo-*F*	*p*	MS	Pseudo-*F*	*p*	MS	Pseudo-*F*	*p*
Site	2	7.77 × 10^−10^	2.41	0.110	0.027	1.22	0.314	0.0020	1.00	0.379	0.0011	3.98	**0.039**
(D, M) vs. S	1	1.49 × 10^−9^	4.82	**0.038**	0.050	2.38	0.134	0.0030	1.54	0.230	0.0021	7.74	**0.013**
Residual	19	3.23 × 10^−10^			0.022			0.0020			2.74 × 10^−4^		

**Table 3:** PERMANOVAs testing the effects of site (Danum [D], Maliau [M], SAFE [S]) on multi-variate lichen community structure, in terms of (a) taxonomic and (b) functional groups, and (c) functional trait composition, in terms of trait classes, including planned comparisons between old growth (D, M) and logged (S) sites. Significant Monte Carlo *p* values are highlighted in bold.

		(a) Taxonomic Groups			(b) Functional Groups			(c) Trait Classes		
Source of Variation	df	MS	Pseudo-*F*	*p*	MS	Pseudo-*F*	*p*	MS	Pseudo-*F*	*p*
Site	2	3569.70	4.69	**<0.001**	2301.50	5.70	**<0.001**	168.14	6.02	**<0.001**
(D, M) vs. S	1	6164.00	7.98	**<0.001**	4140.10	10.18	**<0.001**	307.82	11.01	**<0.001**
Residual	19	761.76			403.77			27.93		

**Table 4:** Contributions of principal lichen (a) taxonomic groups, (b) functional groups and (c) trait classes to differences in multivariate community structure and trait composition between old growth (Danum [D], Maliau [M]) and logged (SAFE [S]) sites. (D,M), S: overall mean abundance in old growth and logged sites, respectively; ${\bar{\delta }}_{i}/\mathrm{SD}\left(\delta_{i}\right)$ = average group contribution to group dissimilarity divided by standard deviation of contributions; ${\bar{\delta }}_{i}\%$ = percent contribution of group to overall between-group dissimilarity. Only the most important taxa (${\bar{\delta }}_{i}\%\;\;$≥ 3%) are shown.

	Group	(D,M)	S	${\bar{\delta }}_{i}/\mathrm{SD}\left(\delta_{i}\right)$	${\bar{\delta }}_{i}\%$
(a)	Taxonomic groups				
	*Cryptothecia*	1.38	51.50	1.99	10.33
	*Eschatagonia*	46.31	9.83	1.59	7.95
	*Graphidaceae* (thelotremoid)	25.19	3.83	1.64	6.94
	*Herpothallon*	39.5	20.17	1.15	6.31
	Graphidaceae (lirellate)	14.56	3.17	1.58	6.16
	*Phyllopsora* and *Krogia*	28.5	7.83	1.59	5.74
	*Anthracothecium* and *Pyrenula*	8.25	2.67	1.48	4.81
	*Biatora*	2.31	5.33	1.10	3.77
	*Leptogium*	2.81	2.67	1.22	3.66
	*Coccocarpia*	3.19	0.17	1.31	3.41
	*Micarea* s.lat.	3.56	1.33	1.08	3.36
	Unidentified crusts	2.25	3.50	1.09	3.30
	*Myeloconis*	1.06	4.17	1.05	3.27
(b)	Functional groups (trait combinations)				
	crustose, thin, ecorticate, prothallus fimbriate	1.38	51.50	2.03	11.33
	squamulose chlorolichen, no hypothallus	46.88	9.83	1.62	8.82
	crustose, apothecia thelotremoid	25.19	3.83	1.67	7.67
	crustose byssoid + apothecia	17.88	2.83	1.90	7.62
	crustose byssoid, thick, sterile, without fimbriate prothallus	39.50	23.50	1.19	7.02
	crustose, apothecia lirellate	15.62	3.17	1.65	6.88
	crustose, perithecia melanised	9.56	2.67	1.49	5.61
	squamulose, Chlorolichen, with hypothallus	11.12	5.00	1.50	5.21
	crustose sterile, coloured pigments exposed	2.31	7.33	1.27	4.63
	foliose cyanolichen, wet: much swelling	2.81	2.67	1.23	4.09
	crustose apothecioid	9.06	12.83	1.10	3.82
	foliose cyanolichen, wet: little swelling	3.19	0.17	1.33	3.77
	crustose, fruiting bodies stalked mazaedioid	2.38	1.50	1.02	3.17
	crustose-squamul. with apothecia and hypothallus	2.31	1.50	1.06	3.15
(c)	Trait classes				
	Fimbriate prothallus	0.02	0.21	1.51	10.24
	Soredia and/or isidia/pseudisidia	0.33	0.16	1.47	9.19
	Squamulose growth type	0.17	0.05	1.85	7.22
	Prothallus, but not fimbriate in structure	0.98	0.79	1.41	6.44
	Fruiting bodies absent	0.49	0.64	1.64	6.38
	Byssoid thallus structure	0.27	0.37	1.32	6.23
	Perithecia with thalline cover	0.19	0.24	1.36	5.39
	Unknown Chlorophyta photobiont	0.42	0.36	1.48	5.03
	Crustose growth type	0.81	0.94	1.78	4.51
	Trentepohliaceae photobiont	0.56	0.63	1.45	4.45
	Apothecia round, with visible margins but not thelotremoid	0.14	0.09	1.46	4.23
	Thelotremoid apothecia	0.08	0.01	1.99	3.76

## Data Availability

Voucher specimens for all taxa are preserved in the collection BORH (Universiti Malaysia Sabah, Kota Kinabalu). All observation data are accessible via the Supplementary Materials.

## Supplementary Materials

The following are available online at https://www.mdpi.com/2076-2607/9/3/541/s1, Figure S1: Boxplots of functional Traits of trees, Table S1: Frequencies of taxonomic groups, Table S2: Frequencies of functional groups, Table S3: Lichen traits matrix, Table S4: Tree taxa and traits observations, Table S5: PerANOVA results of site effects on tree traits, Table S6: Results of Indicator Value analysis of lichen data.
