# Supplementary material for: Key Roles of Dipterocarpaceae, Bark Type Diversity and Tree Size in Lowland Rainforests of Northeast Borneo—Using Functional Traits of Lichens to Distinguish Plots of Old Growth and Regenerating Logged Forests
Source: Microorganisms. 2021 Mar 5;9(3):541. doi: 10.3390/microorganisms9030541 (PMC7999027; doi:10.3390/microorganisms9030541)
Supplement: Supplementary file 1 [file microorganisms-09-00541-s001.zip › Table S5_PERANOVAs of site effects on tree traits.docx]

**Supp. Mat. Table 5.** PERANOVAs testing the effects of site (Danum [D], Maliau [M], SAFE [S]) on plot-level diversity (*H’*) of tree (a) bark type and (b) pH category and proportion of (c) large trees, (d) trees with buttresses and (e) dipterocarps, including planned comparisons between old growth (D, M) and logged (S) sites. Significant Monte Carlo *P* values are highlighted in bold.

|  |  | Diversity | | | | | | |  | Proportion | | | | | | | | | | |
| --- | --- | --- | --- | --- | --- | --- | --- | --- | --- | --- | --- | --- | --- | --- | --- | --- | --- | --- | --- | --- |
|  |  | (a) Bark type | | |  | (b) pH category | | |  | (c) Large trees | | |  | (d) Trees with buttresses | | |  | (e) Dipterocarps | | |
| Source of variation | df | MS | Pseudo-*F* | *P* |  | MS | Pseudo-*F* | *P* |  | MS | Pseudo-*F* | *P* |  | MS | Pseudo-*F* | *P* |  | MS | Pseudo-*F* | *P* |
| Site | 2 | 0.85 | 14.45 | **<0.001** |  | 0.071 | 1.29 | 0.299 |  | 0.20 | 33.75 | **<0.001** |  | 0.017 | 2.27 | 0.131 |  | 0.14 | 8.87 | **0.002** |
| (D, M) vs. S | 1 | 1.59 | 25.84 | **<0.001** |  | 0.080 | 1.43 | 0.238 |  | 0.41 | 69.70 | **<0.001** |  | 0.033 | 4.49 | **0.048** |  | 0.28 | 18.62 | **<0.001** |
| Residual | 19 | 0.06 |  |  |  | 0.056 |  |  |  | 0.0061 |  |  |  | 0.0076 |  |  |  | 0.016 |  |  |
