# Supplementary material for: Key Roles of Dipterocarpaceae, Bark Type Diversity and Tree Size in Lowland Rainforests of Northeast Borneo—Using Functional Traits of Lichens to Distinguish Plots of Old Growth and Regenerating Logged Forests
Source: Microorganisms. 2021 Mar 5;9(3):541. doi: 10.3390/microorganisms9030541 (PMC7999027; doi:10.3390/microorganisms9030541)
Supplement: Supplementary file 1 [file microorganisms-09-00541-s001.zip › Table S6_Indicator_Value_Analyses_Results.docx]

**Suppl. Mat. Table S6** Results of Indicator Value analyses for lichen taxonomic groups (a), functional groups (=trait combinations) (b) and functional trait classes (c) for old growth (Danum, Maliau) and logged (SAFE) sites. Multilevel pattern analysis with association function IndVal.g and significance level 1.0. Columns 'A' and 'B' represent positive predictive values ('A') and sensitivity as a bioindicator ('B') in the sense of DeCáceres & Legendre (2009). Significance levels indicated as: '***' 0.001, '**' 0.01, '*' 0.05, '.' 0.1, ' ' 1.

|  |  |  | A | B | stat | p-value |
| --- | --- | --- | --- | --- | --- | --- |
| (a) | Danum |  |  |  |  |  |
|  | *Micarea* s.lat. |  | 0.798 | 0.875 | 0.836 | 0.0069** |
|  | *Gyalideopsis* |  | 1.000 | 0.125 | 0.354 | 1.0000 |
|  | *Sarcographa* |  | 1.000 | 0.125 | 0.354 | 1.0000 |
|  | *Phyllopsora* & *Krogia* |  | 1.000 | 0.125 | 0.354 | 1.0000 |
|  |  |  |  |  |  |  |
|  | Maliau |  |  |  |  |  |
|  | *Opegrapha* |  | 0.8889 | 0.5000 | 0.667 | 0,0345* |
|  | *Trypethelium* |  | 1.0000 | 0.3750 | 0.612 | 0.0836. |
|  | *Flakea* |  | 0.9286 | 0.2500 | 0.482 | 0.2933 |
|  | *Chrysothrix* |  | 1.0000 | 0.1250 | 0.354 | 1.0000 |
|  | *Letrouitia* |  | 1.0000 | 0.1250 | 0.354 | 1.0000 |
|  | *Parmeliella* |  | 1.0000 | 0.1250 | 0.354 | 1.0000 |
|  | *Sporacestra* |  | 1.0000 | 0.1250 | 0.354 | 1.0000 |
|  | *Psoroglaena* |  | 1.0000 | 0.1250 | 0.354 | 1.0000 |
|  |  |  |  |  |  |  |
|  | SAFE |  |  |  |  |  |
|  | *Cryptothecia* |  | 0.9493 | 1.0000 | 0.974 | 0.0001*** |
|  | *Myeloconis* |  | 0.6623 | 0.6667 | 0.664 | 0.2149 |
|  | *Malmidea* |  | 0.8421 | 0.3333 | 0.530 | 0.5700 |
|  |  |  |  |  |  |  |
|  | Danum + Maliau |  |  |  |  |  |
|  | Thelotremoid Graphidaceae |  | 0.9293 | 1.0000 | 0.964 | 0.0001*** |
|  | Lirellate Graphidaceae |  | 0.9019 | 0.9375 | 0.920 | 0.0064** |
|  | *Pyrenula* & *Anthracothecium* |  | 0.8609 | 0.9375 | 0.898 | 0.0456* |
|  | *Coccocarpia* |  | 0.9745 | 0.8125 | 0.890 | 0.0130* |
|  | *Dichosporidium* |  | 0.8974 | 0.3750 | 0.580 | 0.5400 |
|  | *Sclerophora* |  | 1.0000 | 0.3125 | 0.559 | 0.3933 |
|  | *Melanophloea* |  | 1.0000 | 0.3125 | 0.559 | 0.3549 |
|  | *Arthonia* s.lat. |  | 1.0000 | 0.1875 | 0.433 | 0.7494 |
|  |  |  |  |  |  |  |
|  | Danum + SAFE |  |  |  |  |  |
|  | Unidentified crusts |  | 0.9062 | 0.5000 | 0.673 | 0.343 |
|  | *Gyalecta* |  | 1.0000 | 0.4286 | 0.655 | 0.104 |
|  | Caliciaceae “green mazaedia” |  | 0.8966 | 0.4286 | 0.620 | 0.335 |
|  |  |  |  |  |  |  |
| (b) | Danum |  |  |  |  |  |
|  | Lirellate crusts |  | 1.000 | 0.125 | 0.354 | 1.000 |
|  | Foliose Chlorolichens |  | 1.000 | 0.125 | 0.354 | 1.000 |
|  |  |  |  |  |  |  |
|  | Maliau |  |  |  |  |  |
|  | Fruticose-filamentuos |  | 0.800 | 0.500 | 0.632 | 0.0682. |
|  | Crusts, perith. with thallus cover |  | 1.000 | 0.375 | 0.612 | 0.0851. |
|  | Squamulose cyanolichens |  | 1.000 | 0.125 | 0.345 | 1.0000 |
|  |  |  |  |  |  |  |
|  | SAFE |  |  |  |  |  |
|  | Sterile crusts with fimbriate prothallus |  | 0.9493 | 1.0000 | 0.974 | 0.0001*** |
|  |  |  |  |  |  |  |
|  |  |  |  |  |  |  |
|  | Danum + Maliau |  |  |  |  |  |
|  | Thelotremoid crusts |  | 0.9293 | 1.0000 | 0.964 | 0.0001*** |
|  | Fertile byssoid crusts |  | 0.9266 | 1.0000 | 0.963 | 0.0016** |
|  | Lirellate crusts |  | 0.9080 | 0.9375 | 0.923 | 0.0064** |
|  | Crusts, dark, exposed perith. |  | 0.8776 | 0.9375 | 0.907 | 0.0311* |
|  | Foliose Cyanolichens |  | 0.9745 | 0.8125 | 0.890 | 0.0113* |
|  | Crusts, flat rimless apoth. |  | 1.0000 | 0.1875 | 0.433 | 0.7424 |
|  | Byssoid crust with apoth. in light coloured stroma |  | 0.9024 | 0.4375 | 0.628 | 0.4422 |
|  |  |  |  |  |  |  |
|  |  |  |  |  |  |  |
|  | Maliau + SAFE |  |  |  |  |  |
|  | Crusts with exposed yellow pigments |  | 0.9373 | 0.7143 | 0.818 | 0.0655. |
|  |  |  |  |  |  |  |
| (c) | Danum |  |  |  |  |  |
|  | Lirellae in stroma |  | 1.0000 | 0.125 | 0.354 | 1.0000 |
|  |  |  |  |  |  |  |
|  | Maliau |  |  |  |  |  |
|  | Thelotremoid apothecia |  | 0.7942 | 0.5000 | 0.630 | 0.0613. |
|  | Dark, part. melanized stroma |  | 1.0000 | 0.3750 | 0.612 | 0.0847. |
|  |  |  |  |  |  |  |
|  | Danum + Maliau |  |  |  |  |  |
|  | Thelotremoid apothecia |  | 0.9190 | 1.0000 | 0.959 | 0.0002*** |
|  | Lirellate apothecia |  | 0.9096 | 0.9375 | 0.923 | 0.0046** |
|  | Carbonised perithecia walls |  | 0.8510 | 0.9375 | 0.893 | 0.0500* |
|  | Photobiont: *Rhizonem*a |  | 0.9697 | 0.8125 | 0.888 | 0.0112* |
|  | Light coloured stroma |  | 0.8933 | 0.4375 | 0.625 | 0.4305 |
|  | Apoth. without visible margin |  | 1.0000 | 0.1875 | 0.433 | 0.7504 |
|  |  |  |  |  |  |  |
|  | Maliau + SAFE |  |  |  |  |  |
|  | Exposed yellow pigments |  | 0.9404 | 0.7143 | 0.820 | 0.0614. |
