# Supplementary figures and images for: Key Roles of Dipterocarpaceae, Bark Type Diversity and Tree Size in Lowland Rainforests of Northeast Borneo—Using Functional Traits of Lichens to Distinguish Plots of Old Growth and Regenerating Logged Forests
Source: Microorganisms. 2021 Mar 5;9(3):541. doi: 10.3390/microorganisms9030541 (PMC7999027; doi:10.3390/microorganisms9030541)

(b)

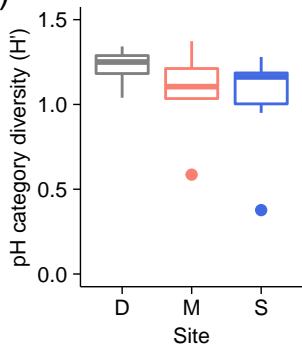

(d)

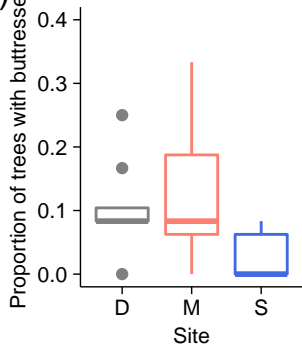

(e)

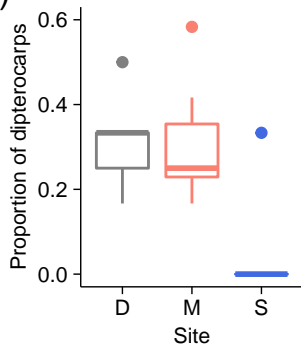

Supplement: Supplementary file 1 [file microorganisms-09-00541-s001.zip › Figure_S1_Functional Traits of Trees_per site_boxplots.pdf]
